# Adsorption of silica oligomers on biomolecules: Structural and dynamical insights for atom probe tomography via classic molecular dynamics simulations

**DOI:** 10.1016/j.csbj.2025.06.004

**Published:** 2025-06-06

**Authors:** Giovanni Novi Inverardi, Lorenzo Petrolli, Francesco Carnovale, Alessio Bartocci, Simone Taioli, Gianluca Lattanzi

**Affiliations:** a Department of Physics, University of Trento, via Sommarive, 14, Trento, 38123, Italy; b INFN-TIFPA, Trento Institute for Fundamental Physics and Applications, via Sommarive, 14, Trento, 38123, Italy; c European Center for Theoretical Studies in Nuclear Physics and Related Areas (ECT*), Bruno Kessler Foundation, Strada delle Tarabelle, 286, Trento, 38123, Italy

**Keywords:** Molecular dynamics, Protein encapsulation, Silica adsorption, Atom probe tomography, Orthosilicates

## Abstract

Atom Probe Tomography (APT) is a spatially-resolved, mass-spectrometric technique, mostly employed in the characterization of metals and alloys. Recently, a novel APT-based protocol has been proposed to resolve the three-dimensional structures of biomolecules, involving the encapsulation of the substrate within an amorphous silica matrix followed by its ablation employing short laser pulses. A critical aspect of this technique lies in the interaction between the silica matrix and the biomolecular substrate, which must keep the native framework of the biomolecule while minimizing the mechanical stresses.

Building on earlier works, here we characterize the adsorption of silica monomers and short oligomers onto biomolecular surfaces via classical Molecular Dynamics (MD) simulations. We observe significant differences in the behavior of the diverse silica species, with the dimers and trimers showing a higher affinity for the substrates. Additionally, unfolded protein domains exhibit an enhanced adsorption efficacy, likely on account of their inherent flexibility and availability of hydrogen-bonding moieties: This apparent affinity dampens their local fluctuations upon interaction with silica, significantly affecting their ensemble dynamics. These findings suggest APT as a suitable technique for the structural characterization of intrinsically disordered regions and the metastable conformational landscapes thereof.

## Highlights

• The adsorption of silica on biomolecules has been assessed by classical MD.
• Silica dimers and trimers effectively coat the surface of biomolecules.
• The interaction with silica is enhanced by flexible and unstructured motifs.
• The silica encapsidation biases the conformational landscape of disordered systems.

## Graphical abstract

**Figure:**
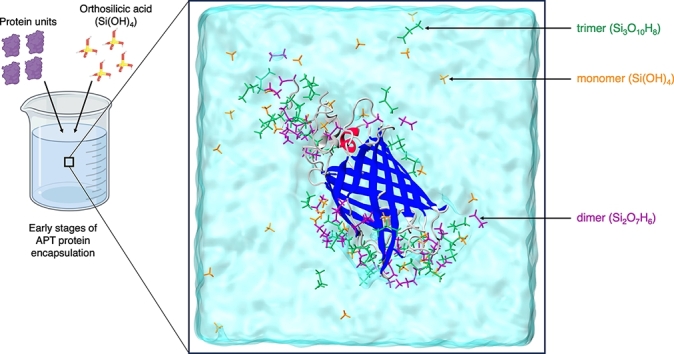

## 1 Introduction

The efficient reconstruction of the high-resolution structures of biomolecules - and the complexes thereof - has significantly advanced our knowledge of their roles and activity. Established techniques, such as X-ray crystallography [1], have been complemented by, e.g., Nuclear Magnetic Resonance (NMR) and cryo-electron microscopy (cryo-EM) [2], offering additional insights into the ensemble and dynamical properties of these systems, provided that a structure at a high-enough quality is obtainable.

Atom Probe Tomography (APT) [3] has been recently discussed as a potential alternative approach. APT is a spatially–resolved, mass spectrometry technique, typically employed in the surface characterization of metals and alloys: In fact, APT is firstly designed for materials capable to withstand cryogenic temperatures, ultra-high vacuum, and intense electric fields, thus requiring experimental conditions that are largely incompatible with the physiological environment of biomolecules in solution.

To address this issue, a novel APT workflow has been developed that involves the dissolution of the biological specimen in a ‘physiological’ (150 mM NaCl) solution of a silica precursor, such as orthosilicic acid (Si(OH)_4_). These molecules are steadily polymerized upon evaporation of the water solvent *via* a sol-gel process, yielding a gel-like network that encapsulates the specimen. The sample is thus dried at room temperature, eventually obtaining an amorphous, glass-like matrix [4].

After an *in situ* lift-out procedure employing a focused ion beam-scanning electron microscope (FIB–SEM) [5], which sharpens the silica matrix into a thin needle, APT is performed on the sample: Briefly, the matrix complex is evaporated atom-by-atom in vacuum under ultra-fast laser pulses, with the ejected species directed towards a sensitive 2D-detector by means of a strong electrostatic field (over 1 V ⋅ Å^−1^) [4]. By associating each signal with spatial coordinates and an atomic mass (derived from the m/z ratio by time-of-flight measurements), a structural profile of the embedded biomolecule is achieved.

A first, proof-of-concept demonstration of this APT pipeline has reported the structure of an Immunoglobuline G (IgG) antibody at a resolution comparable to cryo-EM [4]. Yet, IgG antibodies are mechanically robust and inherently stable towards the stresses associated with the encapsulation procedure, the latter implying that the silica scaffold preserves the native, functional shape of the embedded biomolecule [4], [6]. As a matter of fact, several proteins, enzymes and antibodies are reckoned to retain both their bioactivity and their structural integrity when embedded in sol-gel silica glasses [7], [8], [9], although it is critical that the applicability of the APT protocol is verified over lighter, flexible biomolecules.

To this concern, a thorough microscopic characterization of the silica adsorption process (and the structural implications thereof) by means of, e.g., *in silico* techniques, is called for. In fact, numerical simulations have been successfully applied to model key steps of the standard APT pipeline. For instance, the occurrence of artifacts through the APT reconstruction phase has been assessed by Molecular Dynamics (MD) and Monte Carlo (MC) methods [10], while a mixed approach based on MD and *ab initio* calculations has been recently employed to characterize the APT evaporation mechanism driven by the twofold (laser, drifting) ion field [11], [12].

**Fig. 1:**
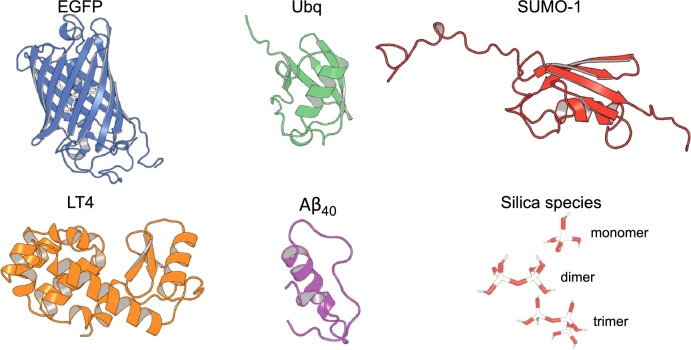
Structures of all systems characterized in this work - namely, EGFP (blue), ubiquitin (green), SUMO-1 (red), lysozyme T4 (orange), and *Aβ*_1−40_ (violet) - and of the silica precursors (i.e., Si(OH)_4_, Si_2_O_7_H_6_, and Si_3_O_10_H_8_).

Building on an earlier work [13], here we make use of classical MD simulations to perform an exhaustive *in silico* assessment of the adsorption of silica onto diverse biomolecular substrates. The systems span both folded and intrinsically-disordered motifs, and involve ubiquitin (Ubq), the small ubiquitin-like modifier 1 (SUMO-1), a member of the Enhanced Green Fluorescent Protein (EGFP) family, lysozyme T4, and a partially folded structure of the amyloid-beta 1-40 (Fig. 1). Additionally, we depict the early stages of the APT protocol and the synthesis of the silica matrix in an effective manner by monitoring the surface affinity of three silica oligomers (i.e., Si(OH)_4_, Si_2_O_7_H_6_, Si_3_O_10_H_8_). We observe that, unlike monomers, silica oligomers dwell on the biomolecular surface, enhancing the silica adsorption, and coalescence without significantly interfering with the structural equilibrium of the folded substrates: Conversely, the deposition of silica oligomers seemingly biases the conformational ensemble of disordered motifs by stabilizing (transient, metastable) folded structures. Furthermore, we infer that the process is enthalpy-driven to a certain extent, and might be enhanced by intrinsically-disordered and/or coiled domains.

## 2 Materials and methods

### 2.1 Crystal structures and MD setup

The crystal structures of all systems characterized in this work - i.e., ubiquitin (Ubq), SUMO-1, EGFP, lysozyme T4, and $A\beta_{1-40}$ - were retrieved from the RCSB Protein Data Bank [14] (entries 1UBQ [15], 1A5R [16], 2Y0G [17], 256L [18], and 1AML [19] respectively). For 2Y0G, missing residues at the amino- (16) and carboxy-terminus (8) were reconstructed employing the Modeller interface [20] in UCSF Chimera [21]. The EGFP chromophore was modeled after the structure in Breyfogle et al. [22], while the coordinates of all silica species - i.e., Si(OH)_4_ (monomer), Si_2_O_7_H_6_ (dimer), Si_3_O_10_H_8_ (trimer) - were obtained from the PubChem database [23] (CID codes: 14942, 61804, and 11966309 respectively).

Three simulation scenarios were thus designed:

• **control**, i.e., the system in a physiological solution;
• **monomeric**, i.e., the system in a physiological solution added with 204 silica monomers;
• **mixed**, i.e., the system in a physiological solution added with 34 silica monomers, 34 dimers and 34 trimers,

thereby fixing the numerical concentration of silica units to approximately 360 mM, consistent with the early stages of the APT protocol [4] (an example setup is shown in Figure S1).

Classical atomistic MD simulations were performed with the Gromacs 2021.5 software [24] in explicit TIP3P [25] water and a 150 mM concentration of monovalent salt. All systems/scenarios were subjected to an equilibration protocol as follows: i) 50000 energy minimization steps by the steepest descent algorithm; ii) a first, 1-nanosecond thermalization of the solvent bath in the NVT ensemble at T = 298 K employing a Berendsen thermostat [26], associated with a coupling constant $\tau_{T}=0.1$ ps; iii) a further, 2-nanosecond volume equilibration in the NPT ensemble at P = 1 bar *via* a Berendsen barostat [26], with $\tau_{P}$ = 2.0 ps and a water compressibility value of 4.5 $\cdot 10^{-5}$ bar^−1^. In all thermalization stages, all heavy atoms in the solute molecules were restrained by a force constant of 1000 kJ/(mol ⋅ nm), and a 1 fs integration time step ($t_{step}$) was applied.

The subsequent, unrestrained NPT equilibration of the systems lasted 5 nanoseconds, followed by several 500-nanoseconds MD replicates, amounting to a total 1.5 μs per scenario. Production simulations were carried out employing a stochastic velocity-rescale thermostat [27] ($\tau_{T}$ = 0.1 ps, T = 298 K), and a Parrinello-Rahman barostat [28] ($\tau_{P}$ = 2.0 ps, water compressibility = 4.5 $\cdot 10^{-5}$ bar^−1^, P = 1 bar), and were associated with an integration time step $t_{step}$ of 2 fs.

The Amber ff14SB [29] and PolCAFF [30] force fields were adopted for the biomolecules and silica species respectively, with Joung-Cheatham corrections for sodium chloride ions [31]. The applicability of the Lorentz-Berthelot combination rules for the calculation of Lennard-Jones interactions ensures the formal cross-compatibility between the Amber ff14sb and PolCA force fields: We shall remark that no specific tuning of the interactions has been performed - which would rely on the availability of experimental data and/or on an *a posteriori* validation anyway. Electrostatic interactions were treated *via* the PME method [32], with a 1.0-nm cutoff applied to both short-range and Van der Waals interactions. All covalent bonds involving hydrogen atoms were constrained by LINCS [33], and long-range dispersion corrections were applied to both energy and pressure calculations.

### 2.2 Analysis protocol

The Gromacs 2021.5 analysis toolkits were employed to compute all mean square quantities, as well as to perform the contact analyses: The latter were carried out by fixing a 6.0 Å threshold distance criterion between heavy atoms, taking into account unique, atom-wise contacts. The solvent-accessible surface area (SASA) was calculated *via* the Gromacs implementation of the algorithm described in Ref. [34], applying a probe radius of 1.4 Å.

The MDAnalysis [35] and SciPy [36] Python libraries were employed to extract significant biomolecular conformations and to perform a hierarchical clustering analysis of the $A\beta_{1-40}$ dynamics. For the latter, a procedure similar to that described in Ref. [13] was adopted. Briefly, each frame in the aggregated trajectory of $A\beta_{1-40}$ is assigned a string array, whereby each residue is mapped to a secondary structure element (e.g., *α*-helices, 3-10 helices, *β*-sheets, etc.) or disordered motif (i.e., coils and turns): Thus, the Hamming distance between these strings is calculated and employed as a similarity metric in a hierarchical clustering analysis based on an average-linkage criterion.

Lastly, iso-density maps of silica occupancy about the systems surface were generated *via* the VMD 1.9.4 interface [37], applying a grid resolution of 1 Å.

## 3 Results

### 3.1 Silica encapsulation of proteins: adsorption patterns and structural determinants

The spontaneous coating of a target biomolecule prior to the laser ablation step plays a pivotal role in the APT experimental protocol. By carrying out unbiased MD simulations at the microsecond time scale, we observe that the silica molecules effectively adsorb on all sampled systems: As shown in Figure S2, the silica encapsulation accounts for a 45/50% reduction in SASA in both the monomeric and the mixed scenarios. Yet, despite a comparable coating efficacy, the adsorption patterns shown by the diverse silica species vary significantly.

**Fig. 2:**
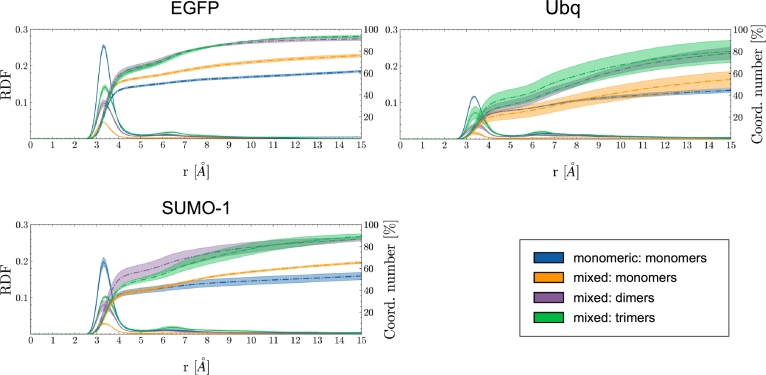
**Left axis**: Radial distribution function (RDF) of silicon atoms about the protein surfaces, averaged over MD replicates. Monomeric scenarios are depicted as blue lines, while contributions from silica monomers, dimers and trimers in the mixed scenarios are shown in yellow, purple and green respectively. **Right axis**: Cumulative distributions of the RDFs (the coordination number is reported as a percentage of the total number of molecules per species), highlighting the different adsorption patterns of the silica species on the surface of EGFP, Ubq, and SUMO-1.

The analysis of the radial distribution functions (RDF) in Fig. 2 shows a consistent pattern, whereby all silica species establish a *first embedding shell* within 4 Å of the protein surface, peaking at approximately 3.5 Å. However, the cumulative distribution of the RDFs reveals a significantly larger fraction of silicon atoms about the substrate in the mixed scenario than in the monomeric scenario, with the silica dimers and trimers exhibiting the strongest affinity. Particularly, the majority of silica dimers and trimers in solution dwells within 15 Å of the surfaces of EGFP, Ubq, and SUMO-1, whereas monomers appear to be homogeneously distributed across the simulation volume in both scenarios.

**Fig. 3:**
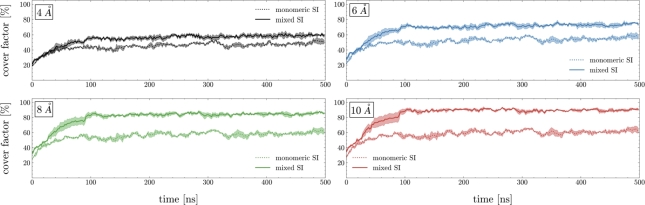
Covering factor of EGFP by silica precursors - i.e., the fraction of silicon atoms within 4 Å (black), 6 Å (blue), 8 Å (green), 10 Å (red) of the protein surface (data are averaged over MD replicates). The monomeric and mixed scenarios are shown as dotted and solid lines respectively. Threshold distance criteria are based on the RDFs shown in Fig. 2.

This observation is further supported by the time evolution of the covering factor shown in Fig. 3, i.e. the fraction of silicon atoms within 4, 6, 8 and 10 Å of the heavy atoms of EGFP (a similar behavior is observed for Ubq and SUMO-1 in Figure S3). The adsorption of silica occurs over approximately 100 nanoseconds in both the monomeric and the mixed scenarios; however, dimers and trimers contribute majorly to the encapsulation process, whereas monomers interact with the protein surface in a somewhat transient manner, consistent with earlier findings [13].

**Fig. 4:**
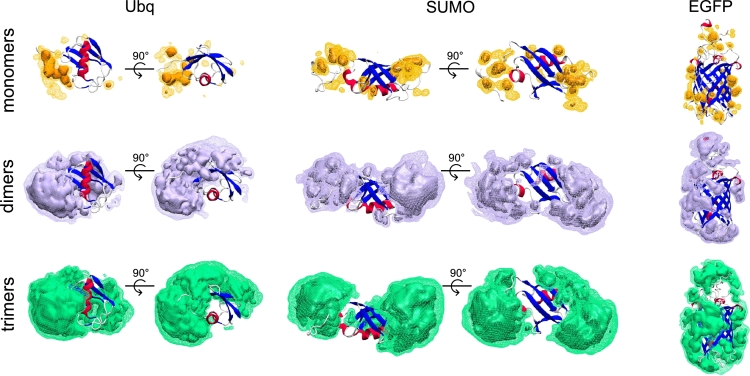
3D density maps of the silica species about the surfaces of EGFP, Ubq and SUMO-1 (expressed in Å^−3^) in the mixed scenarios, highlighting the differences in coating affinity and the inhomogeneous nature of the encapsulation process. Iso-density values are depicted as wireframes (0.10 Å^−3^) and darker regions (0.20 Å^−3^). Silica monomers, dimers, and trimers are shown in orange, purple and green respectively.

Notably, the silica coating shells are not homogeneously distributed about the protein surfaces but rather aggregate into localized clusters of dimers and trimers, as illustrated by the iso-density heatmaps in Fig. 4 (in fact, the tendency of silica to aggregate in solution had been observed both *in vitro* and *in silico* [38], [39]). This inhomogeneity is seemingly driven by a favorable adsorption of silica (clusters) onto flexible and/or unstructured protein domains: For instance, the core *β*-barrel of EGFP is largely devoid of silica molecules, which rather accumulate about its peripheral loops. Similarly, the folded cores of Ubq and SUMO-1 interact less effectively with silica dimers and trimers with respect to their disordered amino- and carboxy-termini, thereby suggesting a correlation between the efficacy of the silica coating and the structural properties of different protein domains.

### 3.2 Heterogeneous coating of proteins by silica: correlations with structural domains

To motivate the apparent selectivity of the coating process, we first verified that the contact analysis between the silica and the proteins recapitulated the outcomes of the 3D iso-density maps (Fig. 4). To this concern, we note that the diverse systems cover various structural motifs: ubiquitin and SUMO-1 serve as proxies for light globular proteins displaying a “mixed” folded core (i.e., involving both *β*-strands and *α*-helices), with SUMO-1 featuring intrinsically-disordered termini; EGFP is a *β*-barrel interspersed with short flexible loops; lastly, lysozyme T4 (LT4) has been included as representative of a fully-folded bundle of *α*-helices.

**Fig. 5:**
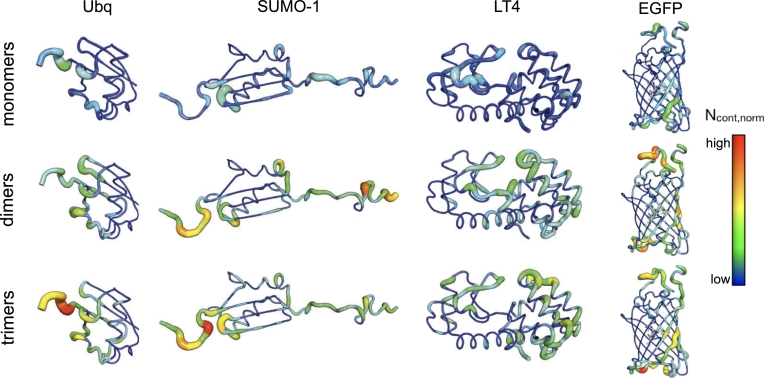
Heatmaps of the number of contacts between all silica species and ubiquitin, SUMO-1, lysozyme T4, and EGFP in the **mixed** scenarios. Contacts are normalized by the number of simulation frames, and averaged across multiple MD replicates. The abundance of contacts is depicted on a color scale ranging from poorly (blue) to densely coated residues (red).

Fig. 5 shows a residue-wise map of the total contacts between the silica species and all systems in the mixed scenarios - normalized by the number of simulation frames, and averaged across MD replicates.

As expected, residues belonging to the unstructured domains of ubiquitin and SUMO-1 (and the neighboring regions thereof) exhibit abundant interactions with the silica species - with the dimers and trimers showing the highest affinity, whereas the monomers establish significantly fewer contacts. This trend is further validated by the interaction pattern observed on the surface of EGFP: Here, the amino- and carboxy-termini and the loops display a higher amount of contacts with the silica than the core barrel, the oligomers showing a consistently higher adsorption efficacy with respect to the silica monomers.

Conversely, contacts between silica and the fully-folded structure of LT4 are established in a more homogeneous fashion, and LT4 exhibits a less pronounced affinity towards silica dimers and trimers, supporting our earlier hypothesis. This behavior might be attributed to the inherent flexibility and availability of hydrogen-bonding donors and acceptors of the disordered domains: In fact, these are typically associated with a larger fraction of hydrophilic and charged residues than fully-structured motifs (see Figure S4), thereby recruiting silica species more effectively.

As regards the surface affinity of silica oligomers, we speculate that this might be driven, to a certain extent, by the establishment of a higher amount of hydrogen-bonding contacts on average (as exemplified by the violin plots in Figure S5), likely on account of a broader conformational versatility and availability of hydroxyl groups with respect to Si(OH)_4_. In fact, the hydrogen-bonding propensity of silicic acid derivatives has been experimentally verified [40].

### 3.3 Silica encapsulation modulates the structural dynamics of disordered domains

**Fig. 6:**
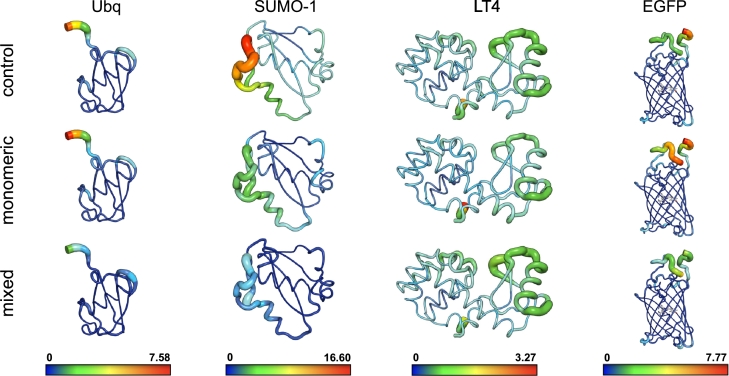
RMS fluctuations of the *α*-carbons of ubiquitin, SUMO-1, lysozyme T4, and EGFP in the *control*, *monomeric*, and *mixed* scenarios respectively, averaged over MD replicates. Colors and thicknesses are scaled proportionally to RMSF values, ranging from low (blue/thin) to high fluctuations (red/thick).

The ability of orthosilicic acid to envelop the surface of proteins without significantly affecting their dynamics has been inferred both experimentally and *in silico* [4], [13]. Similarly, Fig. 6 shows that the RMS fluctuations of the *α*-carbons in all monomeric scenarios are equivalent to (or slightly lower than) those observed in the control trajectories, i.e., in the absence of silica. However, the adsorption of silica oligomers broadly dampens the fluctuations of the target biomolecules in the mixed scenarios. As expected, the influence of silica is more significant on the dynamics of the disordered domains and unstructured moieties, such as the amino- and carboxy-termini of ubiquitin, SUMO-1 and EGFP: This is highlighted by a marked color shift in Fig. 6, further suggesting that the silica adsorption contributes to the structural stabilization of disordered domains from the early stages of the synthesis of the silica matrix. Conversely, the folded helix bundle of lysozyme T4 is minimally affected by the embedding process, as displayed by equivalent RMSF patterns among the three MD scenarios.

Notably, this inhibition of structural fluctuations seemingly biases the selection of locally metastable conformations within the (otherwise degenerate) ensemble of disordered and unstructured moieties. This phenomenon is illustrated better by the observed evolution of the secondary structures in $A\beta_{1-40}$ - i.e., the 40-residue carboxy-terminus of the *Aβ* protein. With the exception of a few transient helical motifs, the conformational ensemble of $A\beta_{1-40}$ is mostly unstructured and characterized by a bundle of coils and loops.

**Fig. 7:**
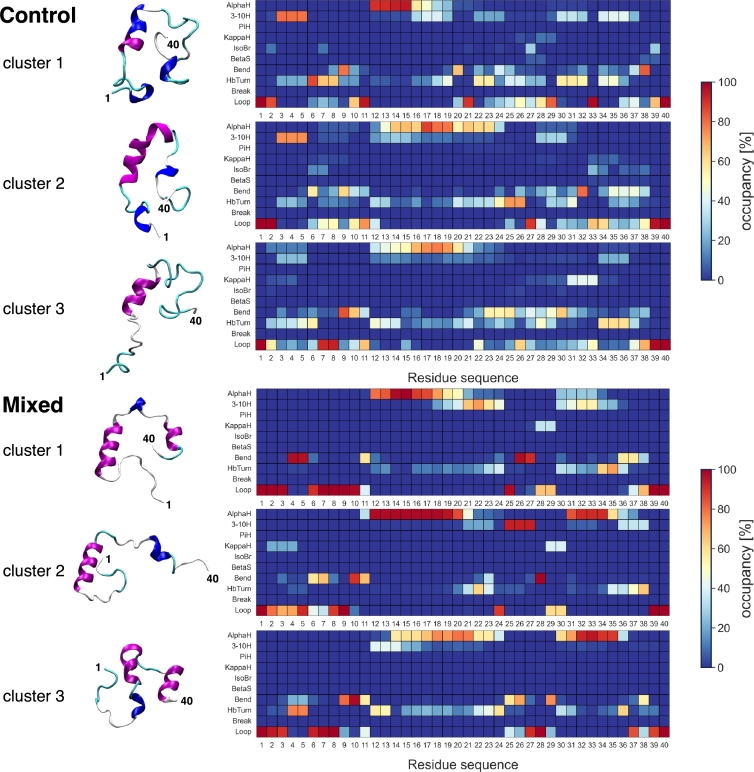
Hierarchical clustering analysis of the dynamics of *Aβ*_1−40_ in the control and mixed scenarios, based on the frame-wise evolution of the secondary structure: The heatmaps illustrate the secondary structure propensity of each residue of *Aβ*_1−40_ within each cluster, categorized into structured (*α*, 3-10, Pi, and K-helices; isolated bridges, and *β*-strands) and unstructured (bends, hydrogen-bonded turns, breaks, and loops/coils) domain motifs. For the purpose of clarity, the three most representative clusters of each scenario are shown, along with a representative conformation.

Fig. 7 shows the outcome of the hierarchical clustering analysis of the aggregated trajectories of $A\beta_{1-40}$ (as described in the Materials and Methods section), highlighting the most significant clusters in both the *control* and the *mixed* scenarios: Particularly, the heatmaps depict the secondary structure propensity (or occupancy) of all residues of $A\beta_{1-40}$ within each cluster, along with a representative conformation.

Several patterns are shared by both scenarios: Overall, $A\beta_{1-40}$ exhibits a high propensity for structural disorder but for a stable helix involving residues 12-24 - despite this motif showing higher occupancy levels in the mixed setup. Yet, a significant discrepancy emerges in the behavior of residues 30-38, which sample a mostly unstructured configurational ensemble in the control scenario, while consistently folding into a helical motif in the mixed scenario. This is in line with earlier circular dichroism and NMR experiments inferring the propensity of Si(OH)_4_ to promote local conformational rearrangements of intrinsically disordered peptides into *α*-helical motifs upon polymerization [41].

## 4 Conclusions

A novel application of Atom Probe Tomography (APT) has been recently developed, featuring the encapsulation of a biomolecule within an amorphous silica matrix followed by a steady ablation through short laser pulses. The evaporated atoms are thus collected by a 2D sensitive grid, thereby allowing the reconstruction of the three-dimensional structure of the target molecule *via* time-of-flight measurements. For this technique to be effective, it is critical that the APT embedding stage keeps the native framework of the biomolecular substrate, while minimizing the mechanical stress.

In this work, we performed a Molecular Dynamics characterization of the adsorption of silica oligomers - namely, the monomer (Si(OH)_4_), dimer (Si_2_O_7_H_6_) and trimer (Si_3_O_10_H_8_) of orthosilicic acid - on diverse biomolecular substrates, effectively depicting the early stages of the synthesis of the silica matrix within the APT protocol. A significant difference is observed between the behavior of silica oligomers and monomers, with the former coalescing about the biomolecular surface and the latter homogeneously distributing in solution. Furthermore, this effect has been verified at diverse concentration ratios of the silica species - as shown in Section “Alternative mixed scenarios of SUMO-1” of the Supplementary Materials.

The analysis of the diverse substrates, characterized by a range of secondary and tertiary structural motifs, suggests that coiled and unfolded domains enhance the adsorption of silica. This effect might be attributable to the intrinsic flexibility and broadly hydrophilic nature of disordered motifs, which effectively behave as favorable binding sites for the silica species. These observations align with earlier works inferring that specific biomolecular features, such as the quality and charge of the amino acid, affect the efficiency and mechanism of biosilicification [42], [43].

Notably, while the embedding process exerts a marginal impact on the conformational ensemble of folded domains, it significantly reduces the fluctuations in coiled regions, seemingly stabilizing metastable helical motifs in intrinsically disordered substrates such as $A\beta_{1-40}$. This apparent structural selectivity, which was similarly suggested in earlier works [41], might deal pivotal insights into the conformational landscape of coiled domains with single-molecule resolution - although further scrutiny would be in order.

Overall, these findings delve into the mechanistic details of the APT embedding protocol, while offering a perspective to employ this technique for the structural characterization of biomolecular substrates at high resolution. Eriksson and co-workers have recently demonstrated that APT accurately detects gold nanoparticles embedded within the silica matrix [44]: Moreover, they have attempted at functionalizing the surface of the nanoparticles with protein substrates prior to silica encapsulation, thereby potentially enhancing the stability of the protocol and/or expanding its scenarios of application - e.g., to the characterization of the microscopic interface between biomolecules and metal surfaces. In fact, APT is rapidly progressing, with additional experimental benchmarks and data likely becoming available in the near future.

## Fundings

This project was funded by the European Union, under 10.13039/100018693Horizon Europe Programme (grant agreement 101046651-MIMOSA). The views and opinions expressed are, however, those of the authors only, and do not necessarily reflect those of the European Union or the European Innovation Council and SMEs Executive Agency (EISMEA). Neither the European Union nor the granting authority can be held responsible for them.

## CRediT authorship contribution statement

**Giovanni Novi Inverardi:** Writing – review & editing, Writing – original draft, Visualization, Validation, Methodology, Investigation, Formal analysis, Data curation. **Lorenzo Petrolli:** Writing – review & editing, Writing – original draft, Supervision, Conceptualization. **Francesco Carnovale:** Writing – original draft, Methodology. **Alessio Bartocci:** Writing – original draft, Supervision, Methodology. **Simone Taioli:** Writing – review & editing, Supervision, Project administration, Funding acquisition, Conceptualization. **Gianluca Lattanzi:** Writing – review & editing, Writing – original draft, Supervision, Project administration, Funding acquisition, Conceptualization.

## Declaration of Competing Interest

The authors declare no conflict of interest.

## Data Availability

The raw data associated with this work are freely available on a Zenodo repository at the link https://zenodo.org/records/14982466.

## Appendix A Supplementary material

MMC Supplementary Material. MMC
