# Supplementary material for: Adsorption of silica oligomers on biomolecules: Structural and dynamical insights for atom probe tomography via classic molecular dynamics simulations
Source: Comput Struct Biotechnol J. 2025 Jun 6;27:2537–43. doi: 10.1016/j.csbj.2025.06.004 (PMC12197878; doi:10.1016/j.csbj.2025.06.004)
Supplement: MMC — Supplementary Material. [file mmc1.pdf]

Supporting Information

Adsorption of Silica Oligomers on Biomolecules: Structural and Dynamical Insights for Atom Probe Tomography via classic Molecular Dynamics Simulations

Giovanni Novi Inverardi,[†,‡] Lorenzo Petrolli,[†,‡] Francesco Carnovale,[†,‡] Alessio Bartocci,[†,‡] Simone Taioli,[¶,§] and Gianluca Lattanzi[*,†,‡]

†*Department of Physics, University of Trento, Via Sommarive 14, I-38123 Trento, Italy*

‡*INFN-TIFPA, Trento Institute for Fundamental Physics and Applications, Via Sommarive 14, I-38123 Trento, Italy*

¶*European Centre for Theoretical Studies in Nuclear Physics and Related Areas (ECT*), Bruno Kessler Foundation, Trento, Italy*

§*Faculty of Applied Physics and Mathematics, Gdańsk University of Technology, Gdańsk, Poland*

E-mail: gianluca.lattanzi@unitn.it

System setup

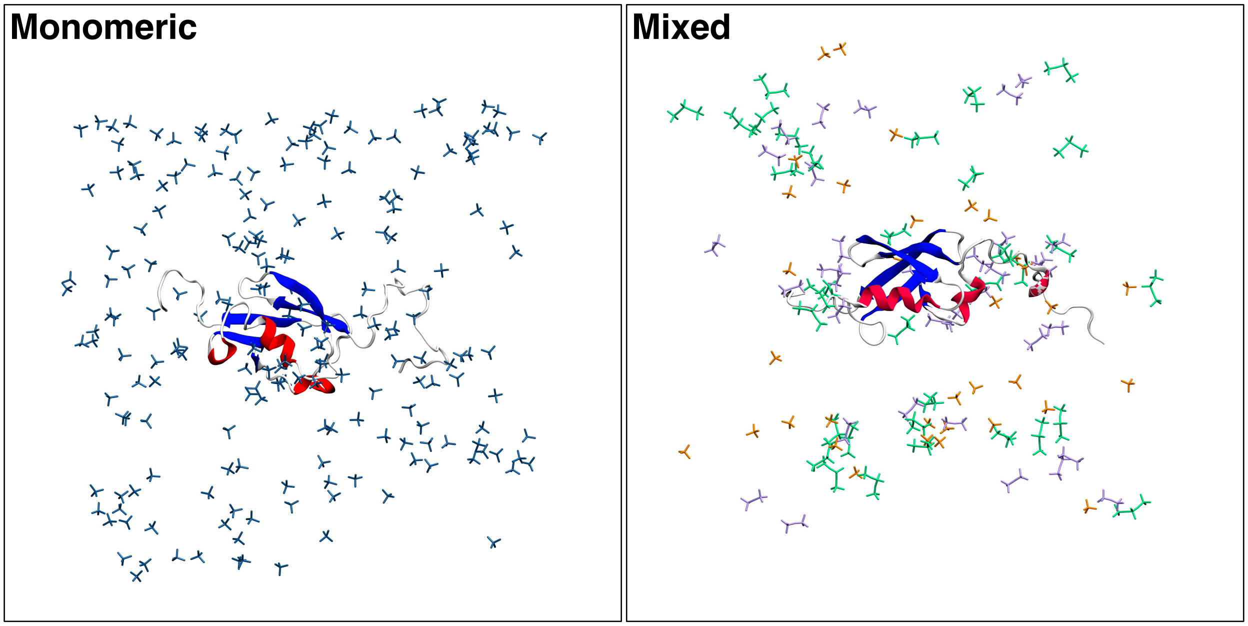

Figure S1: Starting configuration of the monomeric and mixed scenarios of SUMO-1 (similar setups have been adopted for Ubq, SUMO-1, lysozyme T4 and $A\beta_{1-40}$ alike): For the latter, silica monomers are colored in orange, dimers in purple and trimers in green.

Data analysis

Solvent accessible surface area (SASA)

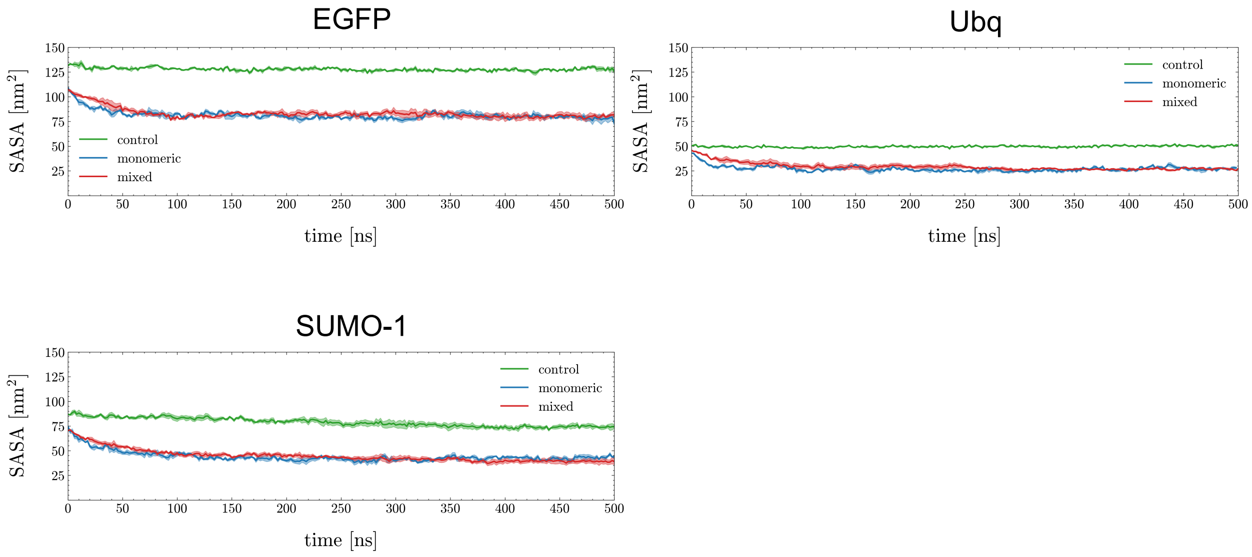

Figure S2: Solvent-accessible surface area (SASA) of EGFP, ubiquitin, and SUMO-1 in the control, monomeric, and mixed scenarios - data are shown as averages of MD replicates.

Covering factor

The covering factor (CF) is defined as the overall fraction of silicon atoms within a threshold distance from a substrate surface.

Ubiquitin

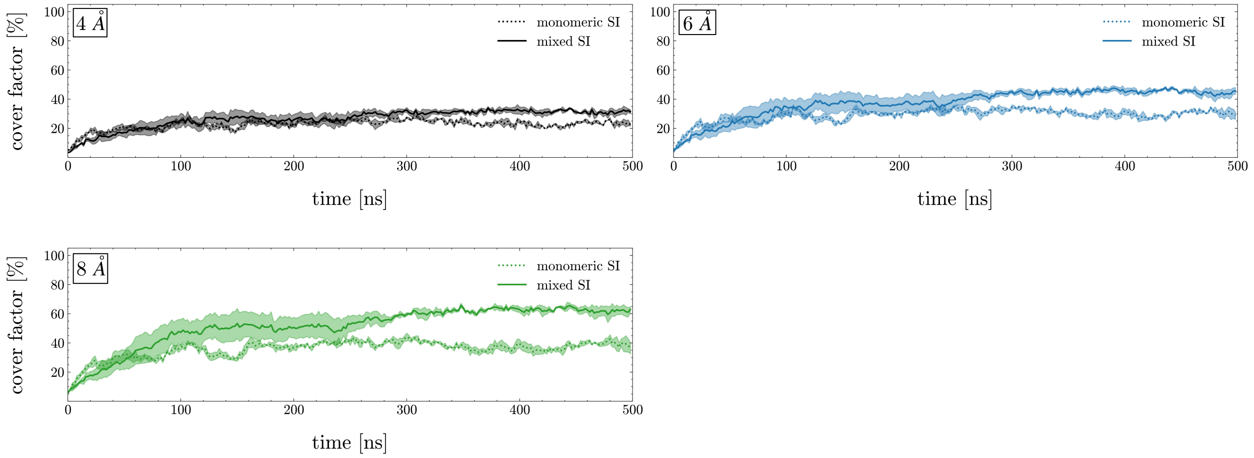

SUMO-1

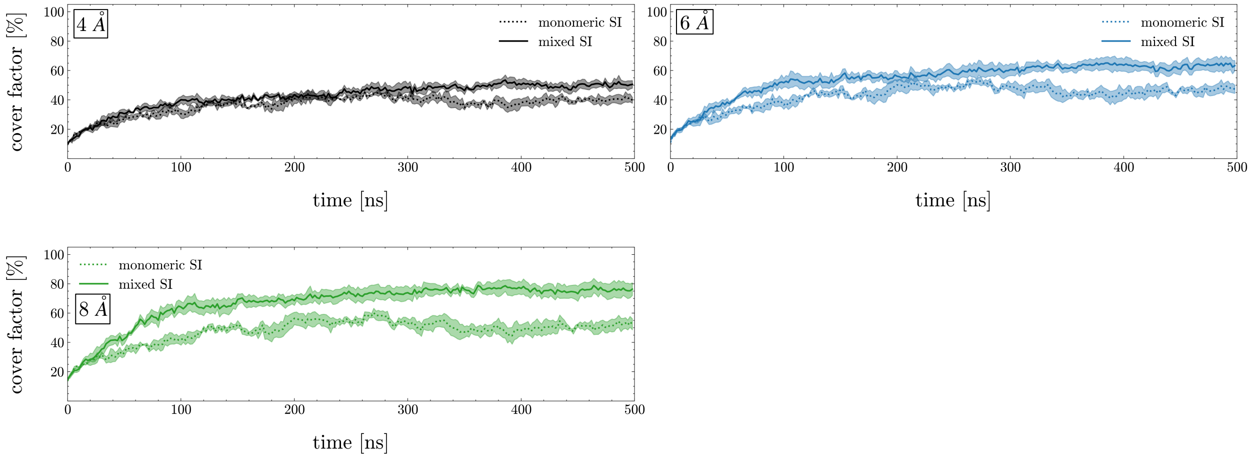

Figure S3: Covering factor - expressed as percentage of silicon atoms - calculated within 4 (black), 6 (blue), and 8 (green) Å from the surface of (**top**) ubiquitin and (**bottom**) SUMO-1 respectively, in the monomeric (dotted lines) and mixed (solid lines) scenarios.

System composition

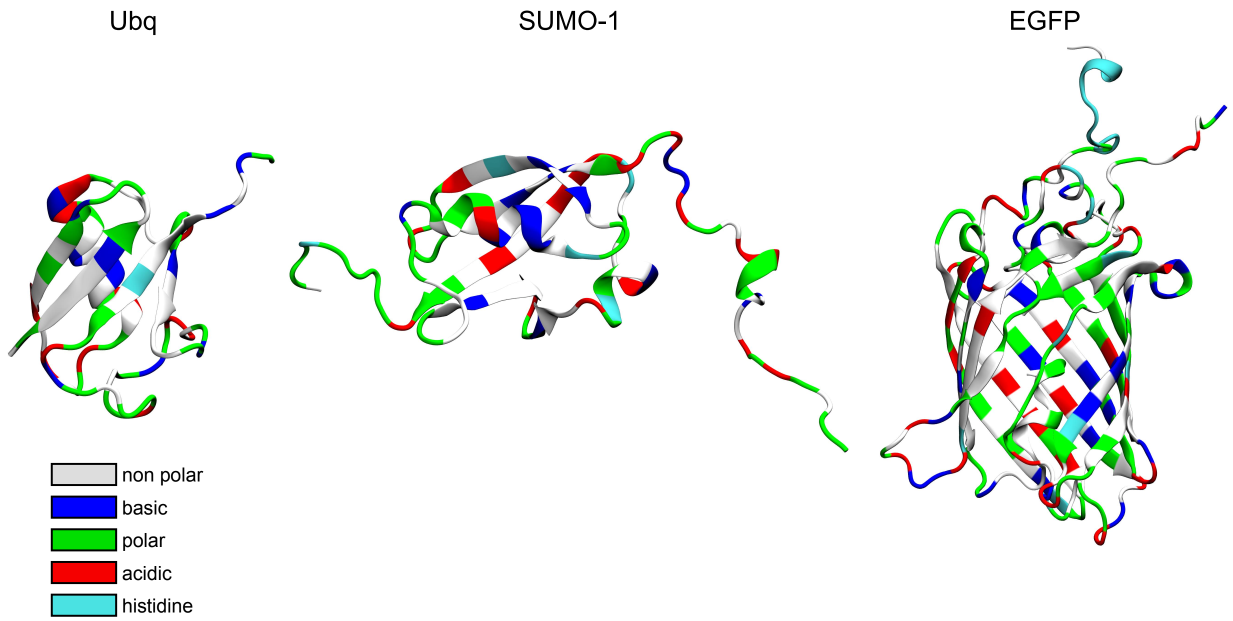

Figure S4: Cartoon representations of Ubiquitin (left), SUMO-1 (middle), and EGFP (right), displaying their composition in terms of hydrophobic (white), polar (green), basic (blue), and acidic (red) residues, and histidines (aquamarine). This illustration was generated *via* the VMD 1.9.4 graphical interface.

Hydrogen bonding propensity

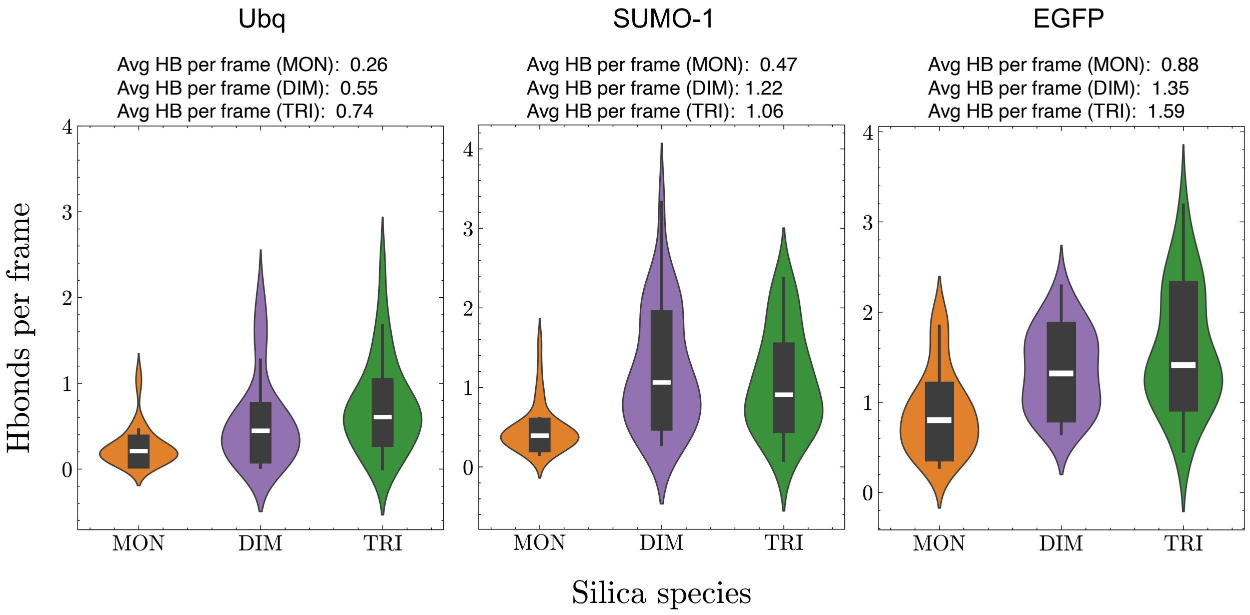

Figure S5: Violin plots representing the number of hydrogen bonds per frame (HBpf) between each silica species - i.e., $Si(OH)_4$ (orange), $Si_2O_7H_6$ (purple), and $Si_3O_{10}H_8$ (green) - and Ubiquitin (left), SUMO-1 (middle), and EGFP (right) in the mixed scenario. The lower and upper edges of the black boxex refer to the first and third quartiles of the HBpf histogram distribution, while the white lines represent the median values. The average values of the HBpf distributions are reported per silica species on top of each plot.

Alternative mixed scenarios of SUMO-1

To verify whether the concentration of silica species (that is, a 1:1:1 ratio between the concentration of silica monomers, dimers, and trimers) affects their adsorption behavior, an alternative mixed scenario was created, where the 204 silicon atoms have been equally split between 23 trimers, 34 dimers and 67 monomers: As such, the concentration of silica monomers is twice the concentration of dimers, and three times the concentration of trimers. We thus performed three 500-ns MD replicates of SUMO-1, taking advantage of its heterogeneous structural framework, in the alternative mixed setup. Results are reported in figures S6 to S9, clearly showing that the adsorption behavior of the silica oligomers is seemingly unaffected by their relative ratio.

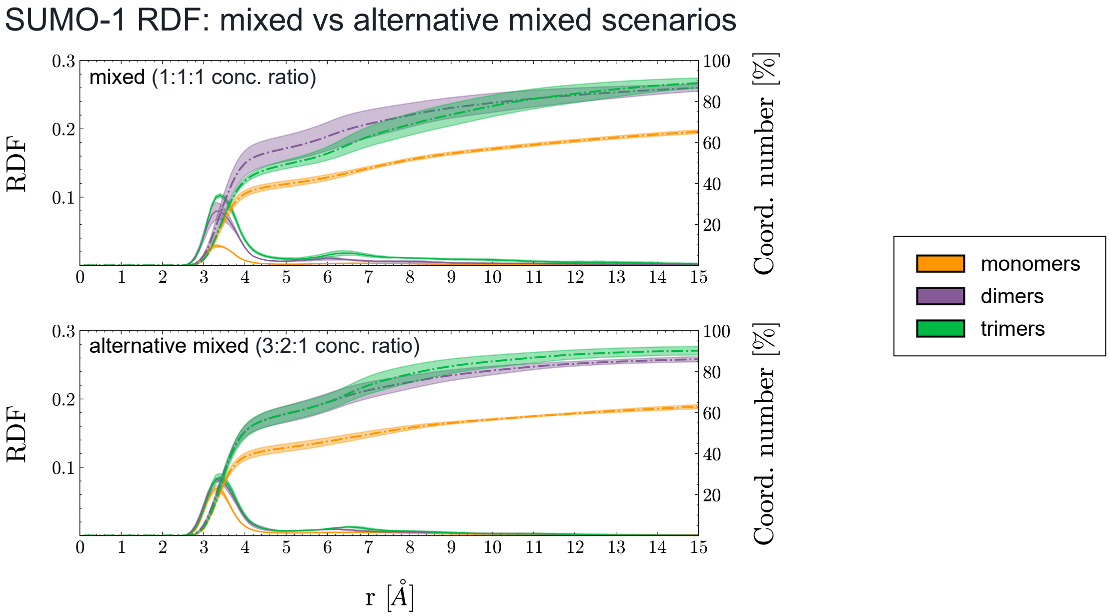

Figure S6: Radial distribution function (RDF) of silicon atoms about the surface of SUMO-1, averaged over MD replicates, and cumulative distributions of the RDFs (the coordination number is reported as a percentage of the total number of molecules per species). Contributions from silica monomers, dimers, and trimers in the mixed (top) and alternative mixed scenarios of SUMO-1 (bottom) are shown in yellow, purple and green respectively, highlighting a similar outcome in the two systems.

SUMO-1 covering factor: mixed vs alternative mixed scenarios

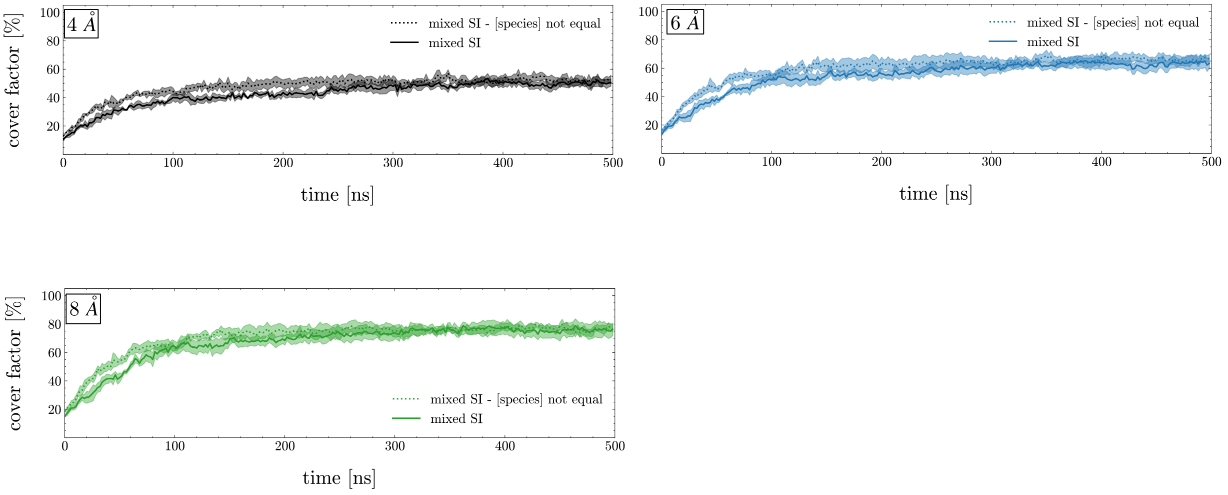

Figure S7: Covering factor - expressed as percentage of silicon atoms - calculated within 4 (black), 6 (blue) and 8 (green) Å from the surface of SUMO-1, in the mixed (solid lines) and alternative mixed (dotted lines) scenarios respectively.

SUMO-1 3D density maps: alternative mixed scenario

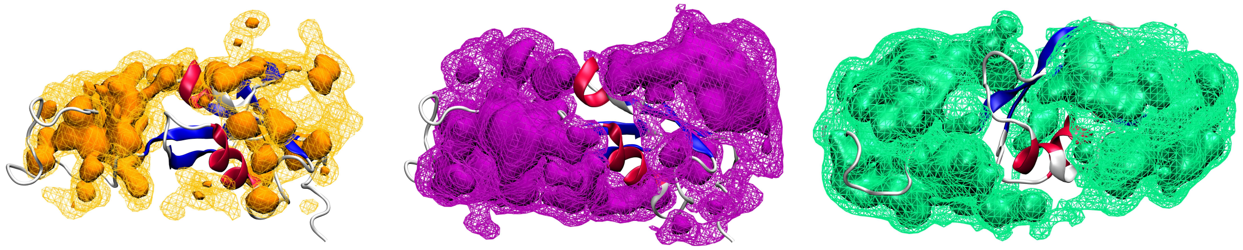

Figure S8: 3D density maps of the silica species about the surface of SUMO-1 (expressed in Points.$Å^{-3}$) in the alternative mixed scenario. Iso-density values are depicted as wireframes (0.10 Points.$Å^{-3}$) and darker regions (0.20 Points.$Å^{-3}$). Silica monomers, dimers, and trimers are shown in orange, purple and green respectively.

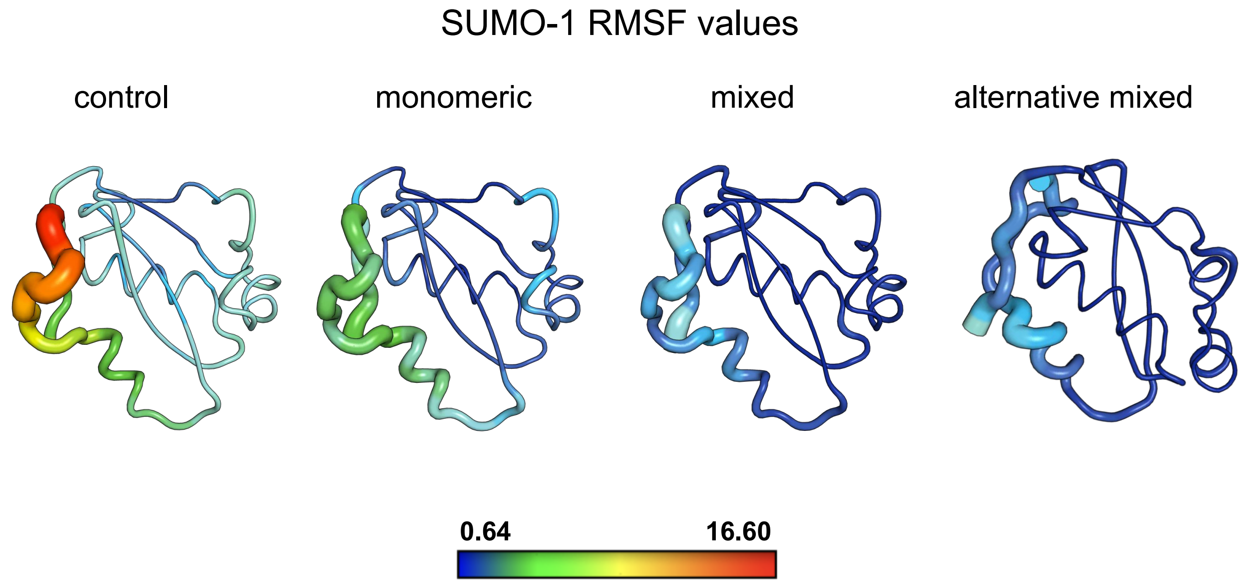

Figure S9: RMS fluctuations of the α-carbons of SUMO-1 in the control, monomeric, mixed and alternative mixed scenarios respectively, averaged over MD replicates. Colors and thicknesses are scaled proportionally to RMSF values, ranging from low (blue/thin) to high fluctuations (red/thick).
